# Advancing Telemedicine Using Smart Insulin Pens with Continuous Glucose Monitoring and Telecommunication Systems: A Case Series

**DOI:** 10.3390/jcm14061794

**Published:** 2025-03-07

**Authors:** Lakshmi G. Singh, Chikara Gothong, Garrett I. Ash, Reynier Hernandez, Elias K. Spanakis

**Affiliations:** 1 Division of Endocrinology, Baltimore Veterans Affairs Medical Center, Baltimore, MD 21201, USA; lakshmi.singh@va.gov; 2 Department of Pharmacy, Baltimore Veterans Affairs Medical Center, Baltimore, MD 21201, USA; 3 Division of Endocrinology, Diabetes, and Nutrition, University of Maryland School of Medicine, Baltimore, MD 21201, USA; 4 Veterans Affairs Connecticut Healthcare System, West Haven, CT 06516, USA; garrett.ash@yale.edu; 5 Department of Internal Medicine, Yale School of Medicine, New Haven, CT 06510, USA; 6 Department of Biomedical Informatics and Data Science, Yale School of Medicine, New Haven, CT 06510, USA

**Keywords:** smart insulin pen, telemedicine, diabetes mellitus, glycemic control

## Abstract

**Background:** Multiple daily injections (MDIs) have been a mainstay for insulin delivery by persons with type 1 diabetes mellitus (T1DM). “Smart” insulin pens (SIPs) offer several advantages over traditional insulin pens, such as a memory function, bolus calculator, and reminders for patients to take their insulin. SIPs can integrate with CGM, allowing for the collection of accurate insulin and glucose data, which can integrate into combined reports. Using these technologies along with telecommunication modalities provides an infrastructure to improve the way in which healthcare can be delivered to those with diabetes. **Methods:** Four cases of uncontrolled T1DM managed by MDIs (and not insulin pumps) and deemed to have plateaued in their management were selected to retrospectively review to identify potential advantages of SIP/CGM along with telemedicine as a method of care delivery. **Results:** This case series revealed potential benefits of this model of care delivery, such as the ability to identify dysglycemia patterns not discernible prior to the use of SIP/CGM, use combined reports as a visual education tool to provide targeted insulin and dietary education, and improve patient engagement in diabetes self-care behaviors. **Conclusions:** We described the benefits of using SIPs and CGM technologies along with telecommunication solutions, as a novel concept for a comprehensive telemedicine system, to improve management of glycemic control and diabetes self-management capabilities.

## 1. Introduction

The incidence of type 1 diabetes mellitus (T1DM) has risen over the past 5 decades with an estimated 15 cases per 100,000 people per year [1]. Mainstays of treatment have been exogenous insulin delivered via a multiple daily injection (MDI) regimen or continuous subcutaneous insulin infusion (CSII) [2]. With advancements in diabetes technology, the use of continuous glucose monitoring (CGM) systems is expanding among patients with T1DM [2]. While CSII is also increasing, many individuals with T1DM are still managed by MDI regimens [3,4,5]. Persons on MDI regimens may experience barriers which affect their insulin adherence and glycemic control, such as fear of hypoglycemia, lack of adequate injection instructions, medication regimen complexity, among others [6,7]. The advent of insulin pens provided a more advantageous method of insulin delivery compared to vials with simpler injection preparation [8], easier administration, and greater accuracy [9]. However, traditional insulin pens lack a memory function to record information regarding insulin usage. Therefore, providers depend on patient self-reports of insulin use to adjust regimens under the presumption of adherence.

In 2007, “smarter” insulin pens (SIPs) were developed with memory capability to store information on date, time, and amount of insulin administered. Additionally, SIPs with Bluetooth connectivity automatically transmit insulin data to a compatible smartphone application [10], making the data accessible for patients or healthcare professionals (HCPs). In 2017, InPen (Medtronic, Northridge, CA, USA) was FDA cleared for use in the United States [11]. Notable features include missed dose reminders for insulin, reminders to check blood glucose, and a bolus calculator, which helps to provide more precise insulin recommendations as it can account for insulin-on-board (IOB), blood glucose targets, and adjust the recommended meal-time dosages. Additionally, it can monitor insulin temperature, among other benefits. Using connected SIP devices along with CGM devices allows integration and sharing of insulin and glucose data that HCPs can use to optimize glycemic control. This is achievable using smartphones, the internet, and compatible software applications. Multiple diabetes data management platforms can also provide these combined data in easily viewable reports (i.e., Glooko, Tidepool, Dexcom clarity, Carelink, among others). Combined CGM/SIP reports can also be directly shared with providers from the SIP application itself.

In addition to advancing the way insulin is administered and glucose is monitored, another integral component is to improve the method by which healthcare is delivered. The Veterans Health Administration (VHA) has been a pioneer in the development of new telehealth technologies, with the first case of telemedicine delivery reported in the 1960s performed within the VHA [12,13]. Since 2008, the VHA has developed secure messaging (email through a secured portal), clinical video telehealth (CVT) (video conferencing connecting patients at rural community-affiliated clinics with providers at more distant medical facilities), and VA video connect (VVC) (patients and providers use video technology using their computers or smart devices) to conduct visits from any location.

In this manuscript, we present our experience remotely managing four patients with T1DM and elevated HbA1c using a comprehensive model of telemedicine for patients with diabetes. These four individuals were selected as they all had T1DM, were managed by MDIs (and not insulin pumps), and all of them had challenges preventing them from achieving improvements in glycemic control and were deemed to have plateaued in therapeutic interventions. They used the InPen (Medtronic Diabetes, Northridge, CA, USA) SIP for insulin administration and data collection, the Dexcom G6 CGM system (Dexcom, Inc., San Diego, CA, USA) for glucose monitoring and data collection, software applications to integrate and combine these data, and telecommunications (VVC, secure messaging, telephone) to conduct visits. Patients were followed up every few weeks following SIP initiation as clinically indicated by their diabetes team as part of routine care. Patients were already on CGM but new to the SIP. All patients were given hands-on training in person for education with a certified diabetes care and education specialist and for input of the calculator settings as recommended by their clinical provider. Telemedicine visits were offered thereafter. Data for CGM metrics are reported based on consensus recommendations [14]. The SIP therapy settings are detailed in Supplementary Table S1.

All patients were provided these technologies and offered telemedicine visits as part of routine diabetes care management and not through a clinical trial or study. Ethical review and approval were waived for this study due to local determination by the VA Research and Development Committee and IRB that this does not qualify as research and does not require consent. Local guidance for sharing case reports was followed; data were not collected for the purpose of this manuscript and were only reviewed after patients had completed their visits with their provider as part of standard of care.

## 2. Case Study Review

### 2.1. Case 1

Therapeutic goals achieved: (1) reduced hyperglycemia; (2) collected objective insulin data for evaluation of adherence; (3) used data to educate and promote behavioral change.

The patient was a 34-year-old male with T1DM without micro- or macrovascular complications, exhibiting significant hyperglycemia with a baseline percentage of time below range (TBR) < 54 mg/dL 0.1%, TBR < 70 mg/dL 2.1%, a percentage of time in range (TIR) 70–180 mg/dL 27.3%, a percentage of time above range (TAR) 181–250 mg/dL of 19.4%, and a percentage of time above range (TAR) > 250 mg/dL 51.3%. Retrieving reliable information from this patient regarding diet and adherence to insulin regimen was an ongoing challenge for years. His healthcare professionals had the impression he was not engaging in the treatment but never connected on this point with the patient. Repeated efforts at education, intensive follow-up, and adjustments to insulin did not result in reaching his goal. Baseline A1c was 10.5%.

Combined CGM/SIP data revealed several issues: missed or delayed pre-prandial insulin aspart doses, administration of insulin aspart when hypoglycemic (without correction), and consumption of high-carbohydrate snacks not covered by prandial insulin leading to severe hyperglycemia. Notably, there were days when no insulin aspart was recorded at all, with CGM tracings revealing sustained severe hyperglycemia. His prescribed total prandial dose based on his report of consuming three meals per day would have dictated ±25 units/day. However, based on SIP data, at times he administered only 12 units/day.

Thus, combined CGM/SIP data helped confirm the disconnect between his prescribed and actual regimen. This streamlined conversations so that more time could be spent addressing behavior change. It also identified gaps in his conception of insulin aspart timing and need based on nutritional intake. For example, he would omit insulin as he underestimated meal carbohydrate content. Combined CGM/SIP reports were also used as a visual educational tool to highlight the glycemic spikes occurring after missed/delayed insulin. At the end of 90 days, although the patient still exhibited significant hyperglycemia on the final visit on his 14-day report with TAR 181–250 mg/dL of 24.5% and TAR > 250 mg/dL 35.7%, his A1c had improved from 10.5% to 8.6%. Despite undergoing standard training protocol for use of the SIP with a diabetes educator and step-by-step manufacturer-recommended training material with return demonstration, the patient’s feedback on the SIP was that initially he felt challenged by the technology, but as he gained more understanding, he generally found it useful. He continued with use at the end of the 90-day period.

### 2.2. Case 2

Therapeutic goals achieved: (1) improved TIR without increase in TBR; (2) improved self-management of DM; (3) educated on relationship between insulin administration and carbohydrate intake.

The patient was a 57-year-old male with T1DM complicated by neuropathy, nephropathy, proliferative diabetic retinopathy, and cerebrovascular accident (CVA) with a history of hypoglycemia and hypoglycemia unawareness who was not reaching the recommended amount of TIR after ongoing follow-up, insulin adjustments, and education efforts. Baseline CGM metrics were TBR < 54 mg/dL 0.2%, TBR < 70 mg/dL 3%, TIR 70–180 mg/dL 37.3%, TAR 181–250 mg/dL 29.9%, and TAR > 250 mg/dL 29.7%. Multiple interdisciplinary team members reported he was ambivalent towards his DM and lacked understanding of the relationship between prandial insulin and carbohydrate intake.

CGM/SIP combined data revealed patterns of nocturnal hypoglycemia due to physician-overestimated basal requirements and hypoglycemia related to administering meal-size insulin doses with snacks. This led to the prescribing of a snack dose and adjustment of his basal insulin. After 14 days of use on essentially the same insulin regimen, the patient’s CGM metrics revealed TBR < 54 mg/dL 0.3%, TBR < 70 mg/dL 1.9%, TIR notably increasing to 62.6%, TAR 181–250 mg/dL 26.9%, and TAR > 250 mg/dL 8.6%. This reflected the patient’s own improvement in self-management of his DM, as evidenced by SIP aspart doses consistent with what was prescribed. As well, he frequently used the SIP’s dose calculator during the first week (83% of all doses) and to an even greater extent as time went on (92% at 90 days) for a mean usage of 87%.

The patient’s final CGM metrics revealed overall improvement from baseline, with TBR < 54 mg/dL 0%, TBR < 70 mg/dL 0.7%, TIR 70–180 mg/dL 51.7%, TAR 181–250 mg/dL 35.6%, and TAR > 250 mg/dL 12%. His overall A1c remained relatively unchanged from 8.3% at baseline to 8.4% at the end of 90 days of use. In this case, the SIP significantly improved the patient’s self-management and engagement in his own DM while also improving his CGM metrics. The patient noted that the use of the SIP helped him to be more attentive to his glucose control and understand his insulin dosing better.

### 2.3. Case 3

Therapeutic goals achieved: (1) reduced and identified causes of hypoglycemia; (2) provided targeted education on insulin use; (3) readjusted basal/bolus insulin dose.

The patient was a 47-year-old male with T1DM complicated by neuropathy, mild non-proliferative retinopathy, a history of significant hypoglycemia, and hypoglycemia unawareness. The patient had intermittent difficulty with maintaining CGM use, leading to transient cessation and monitoring of glucoses by point-of-care (POC) fingersticks (FSs). At visits he often provided these values by memory recall to the HCPs. The volume of data based on the limited frequency of POC-FS and subjective reporting of the values proved to be insufficient to reveal the true severity and etiology of hypoglycemia that he was exhibiting. Upon resuming CGM, baseline metrics were TBR < 54 mg/dL of 11.7%, TBR < 70 mg/dL of 20.3%, TIR 70–180 mg/dL 54.8%, TAR 181–250 mg/dL 20.4%, TAR > 250 mg/dL 4.6%. The baseline CGM report revealed patterns of early daytime hypoglycemia and nocturnal hypoglycemia. The patient worked the night shift and had varying routines on working days versus his days off. Therefore, the exact timing of insulin administration was difficult to be determined by HCPs.

Combined CGM/SIP reports immediately benefited the HCPs as they provided objective data as to when he was administering his prandial insulin. The combined data revealed nocturnal hypoglycemia, some occurring after he inappropriately administered pre-prandial insulin aspart while hypoglycemic (without treatment of hypoglycemia), with other episodes appearing to more inconsistently occur. The reports guided questioning, which revealed the patient was not aware that correction of hypoglycemia before administering insulin for his meals is necessary. Additionally, the patient was administering prandial doses without finishing his meals at work due to insufficient break times. To address this, education was provided on the rule of 15s for treatment of hypoglycemia as well as recommending post-meal administration of insulin aspart when at work, given inconsistent time for breaks leading to the inability to finish meals. Daytime hypoglycemia frequently occurred in the absence of recent insulin aspart injections or active IOB, indicating the patient had lower basal requirements than previously realized. Dangerously, this frequently occurred while sleeping. Ultimately, basal insulin was reduced overall by 33%.

Baseline A1c reduced from 7.7% to 6.9% during this 90-day period, and this is in the setting of less hypoglycemia and as such reflects a true improvement in glycemic control. Patient’s TBR was lowest at 30 days after use; TBR < 54 mg/dL was 1.3%, and TBR < 70 mg/dL was 3.8%. Unfortunately, this increased near the 90-day visit when TBR < 54 mg/dL was 9% and TBR < 70 mg/dL was 15.7%. Patient’s participation in visits declined between 30 and 90 days when there was less frequent interval follow-up than what he previously had (i.e., every 2–4 weeks). He reported having several deaths in the family, which changed his schedule, stress level, and eating habits. He attributed his decreased engagement in his DM and deterioration in glycemic control (including rising TBR) to these stressors. He also experiences total unawareness of hypoglycemia, which unfortunately leads him to be more unintentionally dismissive of his hypoglycemia and can delay treatment since he feels “normal”. He also was not adhering to the previous recommendations and education that initially helped to reduce TBR.

This case demonstrates the significance of patient-related factors that can impact therapeutic inertia, such as the patient possibly lacking awareness and/or being in denial of the severity of his hypoglycemia and the implications of it. While the use of combined CGM/SIP data identified the etiology of his severe hypoglycemia with successful implementation of targeted interventions/education, this case emphasizes the importance of ongoing training and education needs to promote long-standing and sustainable behavioral changes. At the end of the 90-day period, he continued to use the SIP.

### 2.4. Case 4

Therapeutic goals achieved: (1) improved TIR; (2) improved engagement in self-management of DM; (3) improved participation with healthcare team; (4) provided targeted education on need for carbohydrate counting–estimation.

The patient was a 24-year-old male with T1DM without micro-/macrovascular complications with uncontrolled DM. He was diagnosed two years prior with an A1c of 17.5% and was started on a basal-bolus insulin regimen. After 18 months of ongoing attempts to optimize the insulin regimen and provide intensive diabetes self-management and carbohydrate education, his A1c remained well above his goal at 9.8%. He frequently did not attend follow-up appointments, was difficult to contact, would have interruptions in CGM use, and demonstrated inconsistent engagement in comprehensive education on carbohydrate counting. Given his young age, it was a priority to try to optimize the use of available DM technology to improve glycemic control and reduce long-term DM complications. Baseline TBR < 54 mg/dL 0%, TBR < 70 mg/dL 0.4%, TIR 70–180 mg/dL 31.8%, TAR 180–250 mg/dL 29.5%, and TAR > 250 mg/dL 38.4%.

His initial 14-day CGM metrics after starting the SIP use, on essentially the same insulin regimen, revealed improvements in glycemic control with TBR < 54 mg/dL 0%, TBR < 70 mg/dL 0.7%, TIR 70–180 mg/dL 55%, TAR 70–180 mg/dL 27.5%, and TAR > 250 mg/dL 16.7%. His CGM use improved also to 96.3%.

Combined CGM/SIP data revealed hyperglycemic excursions related to mistiming of insulin, insulin under-coverage for high-carb foods (pizza, pasta), or alternatively over-coverage when eating low-/no-carb meals (salads). He was also not covering his snacks with any insulin aspart, leading to hyperglycemic excursions. Trends observed on combined CGM/SIP reports helped underscore the criticality of carbohydrate counting, or at least carbohydrate estimation, to the achievement of his desired glycemic metrics.

The combined CGM/SIP reports were used as a visual educational tool to emphasize what happens when prandial insulin dosing does not match carbohydrate intake. His prandial regimen was adjusted to allow for higher dosing based on carbohydrate meal estimation when consuming higher carb meals, and he was referred for re-engagement in education to move towards a carbohydrate-counting regimen.

In this case, the SIP improved patient engagement with self-management of DM, use of insulin dosing and timing, as well as participation in visits and his healthcare team. At 90 days, his A1c was unchanged at 9.8% at baseline and at 90 days post-SIP use, but it was noted that he spent substantial time off CGM and the SIP due to a change in his smartphone and a lack of compatibility. This led to an increase in hyperglycemia. He did not have CGM metrics at the end of the 90-day period as a result. He resumed CGM and SIP use once he regained compatibility. He had a visit about halfway through this 90-day period, at which time the CGM metrics revealed a TBR < 54 mg/dL 0%, TBR < 70 mg/dL 0.4%, TIR 70–180 mg/dL 50.1%, TAR 180–249 mg/dL 24.3%, and TAR > 250 mg/dL 25.2%, a meaningful improvement in TIR by 18.3% without the expense of increasing TBR.

This patient voiced the highest level of satisfaction of all patients. Upon initial 14-day glycemic improvements, he endorsed immediate tangible benefits with feeling significantly less symptomatic of his severe hyperglycemia, reporting more energy and less polyuria. Furthermore, he and his family felt safer with SIP use as he no longer was exhibiting severe hypo-/hyperglycemic excursions. He also generally felt safer to administer more snack-time and correction doses as the calculator can more precisely recommend insulin dosing, which ultimately helps to lower TAR.

## 3. Summary of Results

All four subjects were male, three were African American, and one was White–Caucasian; ages ranged from 24 to 57, years since diagnosis ranged from 2 to 33, and A1cs prior to the start of SIP use ranged from 7.7% to 10.5%. All patients were managed with insulin glargine for basal insulin and insulin aspart for prandial insulin. Supplementary Table S1 includes the SIP therapy settings.

## 4. Discussion

When patients with DM fail to achieve their glycemic targets, this increases the risk of development of micro- and macrovascular complications and increased risk of morbidity and mortality. As such, it is a priority that novel technologies are explored to overcome this challenge. In this case series, we examined the use of SIPs, CGM devices, and telemedicine to improve glycemic control and self-engagement in poorly controlled T1DM. All four patients were deemed to be good candidates for this multifactorial intervention to use SIPs along with CGM and be followed by a more intensive follow-up strategy using more advanced telemedicine communication options. HCPs were unable to overcome the individual case challenges to achieve their individualized therapeutic goals on standard MDIs and traditional methods of healthcare delivery.

Overall, A1c improved in two patients (Case 2: 1.9% and Case 3: 0.8%) and did not change in the other two. Case 2 had an improvement in TIR of 14.4% after 90 days of SIP use, which is important as each 5% increase in TIR has been associated with clinically meaningful benefits in DM [14]. Associations of TIR with reduced risk of microvascular complications have also been previously evaluated. Beck et al. showed that a reduction in TIR by 10% was associated with an increased hazard rate of progression of retinopathy by 64% and 40% for microalbuminuria [15]. Therefore, improving TIR represents an important therapeutic goal for the management of DM.

The use of SIPs to improve engagement is another important benefit. Previously, it has been demonstrated that in a veteran patient population with DM, 52% of insulin users reported non-adherence [16]. Without objective confirmation of engagement, HCPs may unsafely adjust insulin under the assumption that the patient is following the prescribed regimen. The use of SIPs can improve confidence in managing daily injections, improve diabetes management [17], reduce missed bolus injections, and improve the ability to appropriately adjust insulin dosing [18]. In this case series, patients similarly expressed that SIPs improved engagement due to the availability of reminders, reduced fears of hypoglycemia due to memory function and the bolus calculator (accounting for IOB), and more precise bolus calculations.

In addition to the patient-perceived benefits, critical HCP benefits for SIP use included having a tool to collect accurate information on insulin data in the context of CGM tracings. CGM/SIP data revealed the etiology of glycemic excursions, which was otherwise not definitively clear. The combined reports were also used as a visual educational tool for patients to understand the relationship between insulin and nutritional intake and the overall importance of insulin timing. Furthermore, use of CGM/SIP reports enabled more meaningful and open dialogues with patients to promote behavioral changes in self-management of diabetes. This is an important benefit, as challenges in self-care can lead to emotional frustrations and challenges delaying improvement in glycemic control [19].

While individual patients had varying benefits on glycemic control and self-management, it should be noted that this case series presents data from only the initial 90 days after SIP use. This may represent a short interval of time to observe the impact of SIPs on changes in glycemic control as well as lifestyle and behavioral changes. Also, there are complex patient-related factors that impact therapeutic inertia to improve medication administration habits and implement lifestyle changes [20]. Suggestions to address patient-related factors include scheduling patients more frequently, making information usable and simplified, regularly connecting with interdisciplinary professionals, and considering the use of newer models of care (i.e., telephone or virtual visits) to improve convenience and access [21]. Success of this approach to managing diabetes includes patients’ adherence to the devices and data sharing, accuracy of the data provided (i.e., CGM accuracy), costs of technologies, and accessibility to them by patients and HCPs. Individuals residing in more rural geographical regions or in an underserved or uninsured population may have limited access to advancing technologies [22].

Lastly, patients with poorly controlled diabetes benefit from more regular visits to their HCP for medication adjustments. However, this is challenging for those with limited access or ability to attend frequent in-person clinic visits. Telemedicine offers a more accessible method to provide care and facilitates more frequent encounters. Telemedicine systems for diabetes management have been previously utilized [23,24,25]. However, these systems were often limited by the technology available at the time. For example, glucose data may have been obtained by use of POC-FS and collected by logbook or memory recall, insulin dosing may have been frequently confirmed by subjective patient report, and visits may have been conducted by telephone. During the COVID-19 pandemic, there was a rapid increase in utilization and overall advancement of telemedicine technologies available due to the need to reduce viral transmission risk that could occur in routine in-person outpatient visits. Studies conducted evaluating the impact of the quarantine/lockdown period of the pandemic revealed that glycemic metrics were not negatively impacted in persons with type 1 diabetes and have shown improvements with the use of telemedicine [26,27,28]. The COVID-19 pandemic helped proliferate the use of telemedicine and encourage leveraging available technologies to monitor and manage patients with diabetes. Studies have also demonstrated benefits with the use of SIPs in type 1 and type 2 diabetes management [18,29,30]. Improvements in times-in-range have been observed in type 1 diabetes with the use of SIPs [18,30].

In this case series, patients were managed by a more advanced telemedicine system where CGM devices allowed for the collection of a high volume of glucose data, SIPs enabled the accurate confirmation of insulin dosing, software and diabetes data management platforms enabled the instantaneous and seamless integration of data available at the time of the encounter when needed, and convenient telemedicine options were utilized (VVC and/or secure messaging), which fostered more frequent contacts between HCPs and patients. All these factors helped to improve observed glycemic control and/or self-management behaviors. Three of four patients have continued the use of SIPs along with CGM, one transitioned to AID, and all continue to be followed up by telemedicine.

Future advancements to improve the digital diabetes ecosystem could include integrating all these technologies presented in these case series into one single system where patients subscribe to this care delivery model and are provided with and educated on all these technologies, all designed to make data-sharing for information exchange and communication seamless and efficient. Earlier intervention using advanced technologies could improve glycemic control. Future directions could also take advantage of the growing interest in the use of artificial intelligence for dosing algorithms to guide insulin titrations [31].

## 5. Conclusions

These four cases were selected as part of this case series as they highlight several benefits of SIP use, including the ability to potentially improve glycemic control, adherence, and/or self-engagement in diabetes management in patients with poorly controlled T1DM managed by MDIs and not insulin pumps. Though some patients appeared to benefit more than others, in all cases the use of SIPs along with CGM benefited the HCPs in obtaining accurate and seamlessly integrated CGM/SIP data, leading to a clearer understanding of patients’ adherence, insulin timing, and source of glycemic excursions. The ability to remotely retrieve these data with compatible smart devices and software applications improves efficiency and allows for care to be remotely provided through telehealth communication. This model of care describes an advanced telemedicine platform for diabetes management. This case series also adds to the generally small body of existing literature on SIP use. Further interventional studies are needed examining the use of SIP devices on improvements in glycemic and patient outcomes.

## Data Availability

No new data were created/generated for this case series.

## Supplementary Materials

The following supporting information can be downloaded at: https://www.mdpi.com/article/10.3390/jcm14061794/s1, Table S1: InPen Application Therapy Settings.

## Abbreviations

The following abbreviations are used in this manuscript:

T1DM	type 1 diabetes mellitus
MDI	multiple daily injection
CSII	continuous subcutaneous insulin infusion
CGM	continuous glucose monitoring
VHA	Veterans Health Administration
CVT	clinical video telehealth
VVC	VA video connect
TBR	time below range
TIR	time in range
TAR	time above range
CVA	cerebrovascular accident
FSs	fingersticks
HCPs	healthcare professionals
