# Supplementary material for: Advancing Telemedicine Using Smart Insulin Pens with Continuous Glucose Monitoring and Telecommunication Systems: A Case Series
Source: J Clin Med. 2025 Mar 7;14(6):1794. doi: 10.3390/jcm14061794 (PMC11943169; doi:10.3390/jcm14061794)
Supplement: Supplementary file 1 [file jcm-14-01794-s001.zip › jcm-3469276-supplementary.pdf]

Supplemental Table 1. InPen Application Therapy Settings

	**Baseline**	**14day**	**30day**	**90day**
**Case 1**	**Long acting:**<br>Insulin Glargine<br>20U QAM/24U QPM<br><br>**Rapid-acting:**<br><br>Meal Therapy:<br>Fixed dose<br><br>Insulin Aspart<br>7 U before BK<br>7 U before LN<br>17 U before DN<br>ISF: 50<br>Target BG: 150mg/dL<br>DOA: 4 hours	**Long acting:**<br>Insulin Glargine<br>18U BID<br><br>**Rapid-acting:**<br><br>Meal Therapy:<br>Fixed dose<br><br>Insulin Aspart<br>5.5U before BK<br>5.5U before LN<br>14U before DN<br>2.5U snacks<br>ISF: 50<br>Target BG: 100mg/dL<br>DOA: 4 hours	**Long acting:**<br>Insulin Glargine<br>20U BID<br><br>**Rapid-acting:**<br><br>Meal Therapy:<br>Fixed dose<br><br>Insulin Aspart<br>5.5U before BK<br>5.5U before LN<br>14U before DN<br>2.5U snacks<br>ISF: 50<br>Target BG: 100mg/dL<br>DOA: 4 hours	**Long acting:**<br>Insulin Glargine<br>20U BID<br><br>**Rapid-acting:**<br><br>Meal Therapy:<br>Fixed dose<br><br>Insulin Aspart<br>5.5U before BK<br>5.5U before LN<br>14U before DN<br>2.5U snacks<br>ISF: 50<br>Target BG: 100mg/dL<br>DOA: 4 hours
**Case 2**	**Long acting:**<br>Insulin glargine<br>31U daily<br><br>**Rapid-acting:**<br><br>Meal Therapy:<br>Fixed dose<br><br>Insulin Aspart<br>12U before BK<br>6U before LN<br>12U before DN<br>ISF: 50<br>Target BG: 150mg/dL<br>DOA: 4 hours	**Long acting:**<br>Insulin glargine<br>28U daily<br><br>**Rapid-acting:**<br><br>Meal Therapy:<br>Fixed dose<br><br>Insulin Aspart<br>12U before BK<br>6U before LN<br>12U before DN<br>ISF: 45<br>Target BG: 100mg/dL<br>DOA: 4 hours	**Long acting:**<br>Insulin glargine<br>30U daily<br><br>**Rapid-acting:**<br><br>Meal Therapy:<br>Fixed dose<br><br>Insulin Aspart<br>12U before BK<br>6U before LN<br>14U before DN<br>3U snacks<br>ISF: 45<br>Target BG: 100mg/dL<br>DOA: 4 hours	**Long acting:**<br>Insulin glargine<br>30U daily<br><br>**Rapid-acting:**<br><br>Meal Therapy:<br>Fixed dose<br><br>Insulin Aspart<br>12U before BK<br>6U before LN<br>15U before DN<br>3U snacks<br>ISF: 45<br>Target BG: 100mg/dL<br>DOA: 4 hours
**Case 3**	**Long acting:**<br>Insulin glargine<br>19U daily<br><br>**Rapid-acting:**<br><br>Meal Therapy:<br>Meal-Estimation<br><br>Insulin Aspart<br>4U low carb	**Long acting:**<br>Insulin glargine<br>17U daily<br><br>**Rapid-acting:**<br><br>Meal Therapy:<br>Meal-Estimation<br><br>Insulin Aspart<br>4U low carb	**Long acting:**<br>Insulin glargine<br>17U daily<br><br>**Rapid-acting:**<br><br>Meal Therapy:<br>Meal-Estimation<br><br>Insulin Aspart<br>4U low carb	**Long acting:**<br>Insulin glargine<br>15 units daily<br><br>**Rapid-acting:**<br><br>Meal Therapy:<br>Meal-Estimation<br><br>Insulin Aspart<br>3U low carb

	5U medium carb<br>6U high carb<br>3U snacks<br>ISF: 50<br>Target BG: 150mg/dL<br>DOA: 4 hours	5U medium carb<br>6U high carb<br>3U snacks<br>ISF: 50<br>Target BG: 150mg/dL<br>DOA: 4 hours	5U medium carb<br>6U high carb<br>3U snacks<br>ISF: 50<br>Target BG: 120mg/dL<br>DOA: 4 hours	4U medium carb<br>5U high carb<br>1-2U snacks<br>ISF: 50<br>Target BG: 120mg/dL<br>DOA: 4 hours
**Case 4**	**Long acting:**<br>Insulin glargine<br>18 units daily<br><br>**Rapid-acting:**<br><br>Meal Therapy:<br>Fixed dose<br><br>Insulin Aspart<br>6 units before BK<br>6 units before LN<br>8 units before DN<br>2 units before snack<br>ISF: 50<br>Target BG: 150<br>DOA: 4 hours	**Long acting:**<br>Insulin glargine<br>19 units daily<br><br>**Rapid-acting:**<br><br>Meal Therapy:<br>Fixed dose<br><br>Insulin Aspart<br>5 units before BK<br>6 units before LN<br>8.5 units before DN<br>2 units before snack<br>ISF: 50<br>Target BG: 100<br>DOA: 4 hours	**Long acting:**<br>Insulin glargine<br>21 units daily<br><br>**Rapid-acting:**<br><br>Meal Therapy:<br>Fixed dose t/c meal-estimation<br><br>Insulin Aspart<br>5 units before BK<br>6 units before LN<br>8.5 units before DN<br>2 units before snack<br>ISF: 50<br>Target BG: 100<br>DOA: 4 hours	**Long acting:**<br>Insulin glargine<br>21 units daily<br><br>**Rapid-acting:**<br><br>Meal Therapy:<br>Fixed dose t/c meal-estimation. Referral to CDCES RD<br><br>Insulin Aspart<br>5 units before BK<br>6 units before LN<br>8.5 units before DN<br>2 units before snack<br>ISF: 45<br>Target BG: 100<br>DOA: 4 hours
**U:** units **QAM:** every morning, **QPM:** every evening, **BK:** breakfast, **LN:** lunch, **DN:** dinner, **ISF:** insulin sensitivity factor, **BG:** blood glucose, **DOA:** duration of action for insulin, **BID:** twice daily, **T/C:** to consider<br><br>**Other settings**: time of day settings were turned off for all cases, no patients had the meal therapy option of carb-counting turned on and therefore did not have an insulin to carb ratio set.				
